# Evaluations of the uptake and impact of the Preferred Reporting Items for Systematic reviews and Meta-Analyses (PRISMA) Statement and extensions: a scoping review

**DOI:** 10.1186/s13643-017-0663-8

**Published:** 2017-12-19

**Authors:** Matthew J. Page, David Moher

**Affiliations:** 1 School of Public Health and Preventive Medicine, Monash University, 553 St Kilda Road, Melbourne, VIC 3004 Australia; 2 Centre for Journalology and Canadian EQUATOR Centre, Clinical Epidemiology Program, Ottawa Hospital Research Institute, Ottawa, K1H 8L6 Canada; 3 School of Epidemiology and Public Health, Faculty of Medicine, University of Ottawa, Ottawa, K1H 8M5 Canada

**Keywords:** Reporting, Systematic reviews, Methodology, Quality

## Abstract

**Background:**

The PRISMA Statement is a reporting guideline designed to improve transparency of systematic reviews (SRs) and meta-analyses. Seven extensions to the PRISMA Statement have been published to address the reporting of different types or aspects of SRs, and another eight are in development. We performed a scoping review to map the research that has been conducted to evaluate the uptake and impact of the PRISMA Statement and extensions. We also synthesised studies evaluating how well SRs published after the PRISMA Statement was disseminated adhere to its recommendations.

**Methods:**

We searched for meta-research studies indexed in MEDLINE® from inception to 31 July 2017, which investigated some component of the PRISMA Statement or extensions (e.g. SR adherence to PRISMA, journal endorsement of PRISMA). One author screened all records and classified the types of evidence available in the studies. We pooled data on SR adherence to individual PRISMA items across all SRs in the included studies and across SRs published after 2009 (the year PRISMA was disseminated).

**Results:**

We included 100 meta-research studies. The most common type of evidence available was data on SR adherence to the PRISMA Statement, which has been evaluated in 57 studies that have assessed 6487 SRs. The pooled results of these studies suggest that reporting of many items in the PRISMA Statement is suboptimal, even in the 2382 SRs published after 2009 (where nine items were adhered to by fewer than 67% of SRs). Few meta-research studies have evaluated the adherence of SRs to the PRISMA extensions or strategies to increase adherence to the PRISMA Statement and extensions.

**Conclusions:**

Many studies have evaluated how well SRs adhere to the PRISMA Statement, and the pooled result of these suggest that reporting of many items is suboptimal. An update of the PRISMA Statement, along with a toolkit of strategies to help journals endorse and implement the updated guideline, may improve the transparency of SRs.

**Electronic supplementary material:**

The online version of this article (10.1186/s13643-017-0663-8) contains supplementary material, which is available to authorized users.

## Background

Systematic reviews (SRs) and meta-analyses are an essential resource for healthcare decision-makers [1]. When conducted well, SRs can provide credible and timely data on a range of enquiries, such as which treatments are effective, ineffective or harmful; which tests accurately diagnose a condition and which exposures are associated with health outcomes. However, the value of SRs depends on how well authors have reported what they did, and what they found. If such information is absent or ambiguous, readers cannot judge whether the results of the SR are robust to the methods used, cannot attempt to reproduce the findings and cannot interpret the findings accurately. This can contribute to the failure to implement the findings of SRs into clinical practice [2]. Therefore, transparent reporting of SRs should be considered critically important by authors of SRs [3, 4].

The transparency of SRs and meta-analyses of health research has been called into question on many occasions [5]. The first formal appraisal of SRs with a focus on medicine was performed by Cynthia Mulrow, who identified several poor reporting practices in a sample of 50 medical review articles published between June 1985 and June 1986 [6]. For example, clearly specified methods of identifying, selecting and appraising studies were available in one article only. Transparency was only slightly better in reviews published in 1996, with less than 25% of articles describing how evidence was identified, evaluated or synthesised [7]. In the last decade, transparency of SRs has certainly improved, yet a high amount of suboptimal reporting persists [8].

Improvements in the transparency of SRs in recent years may be attributed to the dissemination of reporting guidelines. Reporting guidelines provide evidence-based recommendations for authors on how to report their research methods and findings clearly [9]. In 1999, an international group of 30 epidemiologists, clinicians, statisticians, editors and researchers developed a reporting guideline for meta-analyses of randomised trials—the QUOROM (QUality Of Reporting Of Meta-analyses) Statement [10]. In 2005, a meeting was convened to update QUOROM to address several conceptual and practical advances in the methodology of SRs and to help overcome several shortcomings identified in an audit of SRs [3]. The guideline was renamed the PRISMA (Preferred Reporting Items for Systematic reviews and Meta-Analyses) Statement, and published in 2009 [11]. It was accompanied by an explanation and elaboration document, which provided detailed guidance for each of the 27 included items, and examples of exemplar reporting [12].

**Fig. 1:**
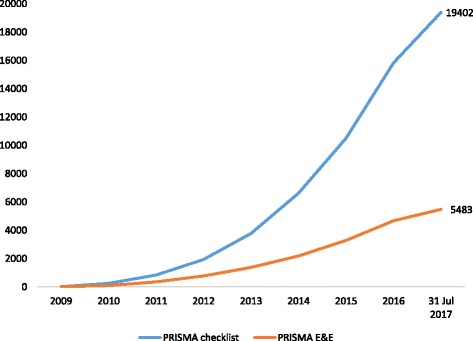
Cumulative number of citations of the PRISMA Statement. Data obtained from Scopus® on 31 July 2017. E&E explanation and elaboration

According to citation data in Scopus®, the PRISMA Statement has had a very high uptake from the biomedical research community (Fig. 1). The checklist paper [11, 13–19] has been cited 19,402 times as of 31 July 2017, and the accompanying explanation and elaboration document [12, 20–23] received 5483 citations by this date. However, not all published SRs cite the guideline; for example, in a random sample of 119 non-Cochrane SRs of therapeutic interventions indexed in MEDLINE® in February 2014, 42 (35%) mentioned the use of the PRISMA Statement [8].

**Fig. 2:**
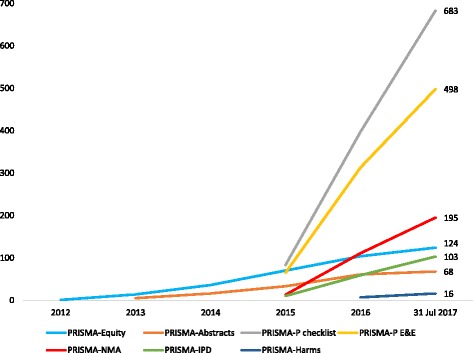
Cumulative number of citations of PRISMA extensions published before 2017. Data obtained from Scopus® on 31 July 2017. E&E explanation and elaboration, IPD individual participant data, NMA network meta-analysis

Since its publication, seven extensions to the PRISMA Statement have been developed to facilitate reporting of different types or aspects of SRs (Table 1). These include the PRISMA-Equity extension [24–26], PRISMA for Abstracts of SRs [27], PRISMA extension for reporting SRs incorporating network meta-analysis [28], PRISMA for SRs and meta-analyses of individual participant data [29], PRISMA for SR protocols [30, 31], PRISMA harms checklist [32] and PRISMA extension for SRs of complex interventions [33, 34]. Citation counts for the PRISMA extensions are much lower than those of the PRISMA Statement, but they have not had the same amount of time to accrue citations (Fig. 2). Also, one should not expect the extensions to receive as many citations, as they are more restricted in scope, meaning that fewer SRs to which the extensions are applicable are published each year. The most cited extension is the checklist paper for PRISMA-P (for SR protocols) [30], which has received 683 citations since its publication in January 2015.

**Table 1:** Scope of the PRISMA Statement and published extensions

Reporting guideline	Year published	Scope of reporting guideline
PRISMA	2009	Reports of systematic reviews and meta-analyses, primarily of randomised trials that evaluate health care interventions [11–23].
PRISMA-Equity	2012	Reports of systematic reviews and meta-analyses with a focus on health equity, defined as the absence of avoidable and unfair inequalities in health [24–26].
PRISMA-Abstracts	2013	Abstracts for all types of systematic reviews, but the emphasis is on systematic reviews of evaluations of interventions where one or more meta-analyses are conducted [27].
PRISMA-Network Meta-Analysis	2015	Reports of systematic reviews that address networks of multiple treatment comparisons [28].
PRISMA-Individual Participant Data	2015	Reports of systematic reviews and meta-analyses of individual participant data. Developed primarily for reviews of randomised trials, but many items apply to other contexts, including reviews of diagnosis and prognosis [29].
PRISMA-Protocols	2015	Protocols for systematic reviews and meta-analyses that summarise aggregate data from studies, particularly those which evaluate the effects of interventions [30, 31].
PRISMA-Harms	2016	Reports of systematic reviews and meta-analyses assessing adverse events (as either a primary or secondary outcome) that are reported in prospective interventional studies or observational studies (with or without a comparison group) [32].
PRISMA-Complex Interventions	2017	Reports of systematic reviews and meta-analyses of complex interventions. Complex interventions are defined as interventions that have ‘multiple components (intervention complexity) and complicated/multiple causal pathways, feedback loops, synergies and/or mediators and moderators of effect (pathway complexity)’ [33, 34].

There are also eight PRISMA extensions that are in development (Table 2). These include extensions for SRs of newborn and child health research and for protocols of such SRs, for SRs of diagnostic test accuracy studies, for rapid reviews, for scoping reviews, for SR search methods, for SRs of traditional Chinese medicine interventions and for SRs of in vivo animal studies.

**Table 2:** Scope of the PRISMA extensions in development

Reporting guideline	Month registered	Scope of reporting guideline
PRISMA-Children	Nov 2014	Reports of systematic reviews and meta-analyses of randomised trials or observational studies of newborn and child health research [155].
PRISMA-Protocol for Children	Nov 2014	Protocols for systematic reviews and meta-analyses of randomised trials or observational studies of newborn and child health research [155].
PRISMA-Diagnostic Test Accuracy	Nov 2015	Reports of systematic reviews and meta-analyses of diagnostic test accuracy studies (i.e. studies of the ability of medical tests to detect a target condition) [156].
PRISMA-Rapid Reviews	Nov 2015	Reports of rapid reviews, including those with analogous terminology (e.g. rapid evidence synthesis, rapid knowledge synthesis) [157].
PRISMA-Scoping Reviews	Dec 2015	Reports of scoping reviews, which are used to map the concepts underpinning a research area and the main sources and types of evidence available [158].
PRISMA-Search	Feb 2016	Reports of literature searches in systematic reviews [159].
PRISMA-Traditional Chinese Medicine	Aug 2016	Reports of systematic reviews and meta-analyses of studies that evaluate Chinese herb medicine or moxibustion [160].
PRISMA-In Vivo Animal studies	To be registered	Reports of systematic reviews and meta-analyses of in vivo animal studies (Manoj M. Lalu, personal communication, June 2017)

Registered PRISMA extensions were identified in the library of reporting guidelines available at the Enhancing the QUAlity and Transparency Of health Research (EQUATOR) Network website (http://www.equator-network.org/library/), on 24 July 2017

It is important to evaluate whether the PRISMA Statement and extensions have achieved what they are designed to do—improve the transparency of SRs. We are aware of two previous SRs that have investigated the *adherence* of SRs to the PRISMA Statement (i.e. the extent to which SRs comply with each item in the statement) [35, 36]. Another SR has examined whether transparency is better in SRs published in journals that *endorse* the PRISMA Statement (e.g. suggest its use in the journal instructions to authors or require that authors submit a PRISMA checklist accompanying their SR) [37]. However, to our knowledge, there has been no attempt to map what other research on the uptake, and impact of the PRISMA Statement and extensions has been done. Also, there has been no attempt to synthesise studies evaluating adherence of SRs published *after* the PRISMA Statement was disseminated. Therefore, we aimed to address these gaps by conducting a scoping review of meta-research studies evaluating the PRISMA Statement and extensions.

## Methods

We did not pre-register the methods of our scoping review, as we are unaware of any register for methodological research of this nature.

We considered articles to be eligible for inclusion if they were an empirical study of any design (e.g. randomised trial, cross-sectional analysis, before-after study), which investigated some component of the PRISMA Statement or extensions (e.g. how often PRISMA is referred to in journal instructions to authors) or which used the PRISMA Statement or one of the extensions for evaluative purposes (e.g. to assess how often SRs adhere to each PRISMA item). We included meta-research studies regardless of language or year of publication. We excluded commentaries, editorials or letters to the editor.

One author (MJP) searched for potentially relevant studies indexed in MEDLINE® from inception to 31 July 2017 (specifically, Ovid MEDLINE® Epub Ahead of Print, In-Process and Other Non-Indexed Citations; Ovid MEDLINE® Daily and Ovid MEDLINE and Versions®). The following search strategy was used to retrieve articles that included the term ‘PRISMA’ (abbreviated or spelled out in full) in the title or abstract of the article:

1. ‘Preferred Reporting Items for Systematic Reviews and Meta-analyses’.ti,ab.
2. PRISMA.ti,ab.
3. 1 or 2.

One author (MJP) screened all titles and abstracts, and any full-text articles retrieved, to determine eligibility. The same author recorded the types of evidence available in the included meta-research studies. Types of evidence were classified as:

- data on SR adherence to the PRISMA Statement or extensions;
- characteristics associated with SR adherence to PRISMA (e.g. journal endorsement, year of publication);
- the frequency of journal instructions to authors referring to the PRISMA Statement or extensions;
- other (e.g. frequency of SR authors who reported using the PRISMA Statement to guide their reporting).

To determine the influence of the PRISMA Statement on the transparency of SRs, we pooled the findings of meta-research studies evaluating how often SRs adhere to the PRISMA Statement. This updates a previous SR which included adherence studies published up to October 2014 [35]. One author (MJP) collected from each meta-research study the following data about the SRs evaluated: focus (e.g. therapeutic, diagnostic), clinical area, language, years of publication and frequencies of SRs adhering to each of the 27 PRISMA Statement items. In some cases, authors of meta-research studies recorded if a particular PRISMA item was fully reported or partially reported in each of the SRs evaluated. In such cases, we recorded only the number of SRs that fully reported the PRISMA item. One author (MJP) contacted study authors to request data on adherence to individual items if these data were not available in the published article (e.g. when study authors reported only the mean number of items that SRs adhered to).

We pooled data on SR adherence to individual PRISMA items across all SRs in the included studies. We noted items that fewer than two thirds (67%) of SRs adhered to and those that are fewer than half of SRs adhered to. We also pooled data on SR adherence to individual PRISMA items in a subset of studies that evaluated SRs published after the PRISMA Statement was disseminated. For this analysis, we analysed studies which included only SRs published in 2010 or later or studies which reported data on a subgroup of SRs published in 2010 or later. We did not contact study authors for this subgroup data. We conducted all analyses in Microsoft Excel.

## Results

### Scoping review of meta-research studies

**Fig. 3:**
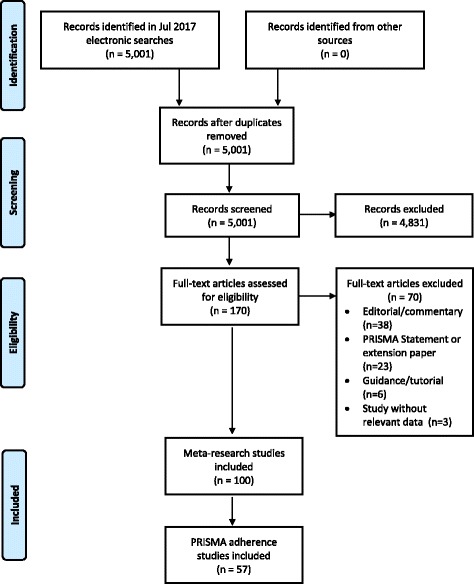
Flow diagram of identification, screening and inclusion of studies

The search of MEDLINE® yielded 5001 citations (Fig. 3). After screening each title and abstract, we retrieved the full text of 170 articles. We excluded 70 of these articles, most of which were editorials or commentaries (reasons for exclusion are listed in Additional file 1: Table S1). One hundred meta-research studies met our inclusion criteria (listed in Additional file 2: Table S2). The studies were published between 2011 and 2017, and more than half were published in 2015 or later (*n* = 59). All of the studies were observational in design; there were 86 cross-sectional analyses, six uncontrolled before-after studies, four surveys of authors and four systematic reviews of meta-research studies.

We recorded 20 different types of evidence available across the included meta-research studies (Table 3). The most common type of evidence available was data on SR adherence to the PRISMA Statement, which was reported in 57/100 (57%) studies. Many of these 57 studies (*n* = 37 [65%]) also investigated characteristics associated with SR adherence to the PRISMA Statement, such as the type of journal, year of publication and article word count. The third most common type of evidence available was data on the frequency of journals referring to the PRISMA Statement or extensions in the instructions to authors (*n* = 18/100 [18%]).

**Table 3:** Types of evidence available in meta-research studies (*n* = 100) evaluating the PRISMA Statement or extensions

Type of evidence available	Frequency of studies
SR adherence to the PRISMA Statement or extensions	
Data on SR adherence to the PRISMA Statement [38–94]	57
Data on SR adherence to a particular item of the PRISMA Statement (e.g. searching item, risk of bias assessment item) [95–99]	5
Data on SR abstract adherence to the PRISMA-Abstracts extension [74, 100, 101]	3
Data on network meta-analysis adherence to the PRISMA-Network Meta-Analysis extension [102]	1
Data on rapid review adherence to the PRISMA Statement [103]	1
Data on SR adherence to draft versions of the PRISMA-Child and PRISMA-Protocols Child extensions [104]	1
Data on SR adherence to reporting standards derived from the PRISMA Statement [8, 105, 106]	3
Data on SR abstract adherence to items derived from the PRISMA Statement [107, 108]	2
Data on individual participant data meta-analysis adherence to items derived from the PRISMA Statement [109]	1
Characteristics associated with SR adherence to the PRISMA Statement	
Association between journal endorsement of the PRISMA Statement and SR adherence to PRISMA [38, 39, 46, 71, 77, 81, 84, 94]	8
Association between factors other than journal endorsement (e.g. type of journal, word count, year of publication) and SR adherence to PRISMA [38, 43, 44, 46–51, 54, 57–62, 64, 66, 68, 70, 71, 73–75, 77, 78, 80, 82–86, 88–90, 92, 94]	37
Mention of the PRISMA Statement or extensions in journal instructions	
Frequency of journals referring to the PRISMA Statement or extensions in the instructions to authors [46, 71, 77, 81, 84, 110–122]	18
Frequency of journals referring to the PRISMA Statement or extensions in the instructions to peer reviewers [112, 123]	2
Other	
Frequency of SR authors who reported using the PRISMA Statement to guide reporting [8, 71, 114, 115, 124]	5
Frequency of editors who are aware of the PRISMA Statement [111]	1
Frequency of inappropriate citation of the PRISMA Statement by authors [125]	1
Association between adherence to the PRISMA Statement and citation of SRs [126]	1
Authors’ perceived barriers and facilitators to use of the PRISMA-Equity extension [127]	1
Authors’ views on what items are most important to report in SRs [128–130]	3
Systematic reviews of meta-research studies evaluating some component of the PRISMA Statement or extensions [35–37, 131]	4

Few studies have evaluated how well SRs adhere to the PRISMA extensions; adherence to PRISMA for Abstracts and PRISMA for Network Meta-Analyses has been examined in three studies and one study, respectively (Table 3). Further, few studies have investigated whether the endorsement of the PRISMA Statement by journals was associated with adherence to PRISMA (*n* = 8/100 [8%]). We did not identify any studies that investigated whether journal endorsement of one of the PRISMA extensions was associated with SR adherence to the extension.

### Evaluations of SR adherence to the PRISMA Statement

Of the 57 studies evaluating SR adherence to the PRISMA Statement [38–94], most were published between 2015 and 2017 (33/57 [58%]), focused on SRs of therapeutic interventions only (45/57 [79%]), evaluated non-Cochrane SRs only (34/57 [60%]) and evaluated SRs written in English only (39/57 [68%]) (Table 4). A total of 6487 SRs were evaluated across all studies; the median (interquartile range) number of SRs evaluated per study was 74 (44-144). The evaluated SRs were published between 1989 and 2016.

**Table 4:** Characteristics of 57 studies evaluating SR adherence to the PRISMA Statement

Characteristic	Summary data
Year of study publication	
2011–2014	24 (42%)
2015–2017	33 (58%)
Focus of SRs evaluated	
Therapeutic interventions (treatment/prevention)	45 (79%)
Diagnostic	4 (7%)
Mix (e.g. some therapeutic, some diagnostic)	6 (11%)
Not specified	2 (4%)
Clinical area of SRs evaluated	
Surgery	14 (25%)
General medicine	5 (9%)
Nursing	5 (9%)
Complementary and alternative medicine	4 (7%)
Other (specific clinical condition)	29 (51%)
Median number of SRs evaluated	74 (44-144)
Median earliest year of publication of SRs evaluated	2005 (2001–2009)
Median latest year of publication of SRs evaluated	2013 (2011–2015)
Journal of SRs evaluated	
Non-Cochrane only	34 (60%)
Both Cochrane and non-Cochrane	22 (39%)
Unclear	2 (11%)
Language of SRs evaluated	
English only	39 (68%)
Chinese only	9 (16%)
Portuguese only	1 (2%)
English and LOE (less than 10% LOE)	6 (11%)
English and LOE (more than 40% LOE)	2 (4%)

Data given as number (percent) or median (interquartile range)

*LOE* language other than English, *SR* systematic review

**Fig. 4:**
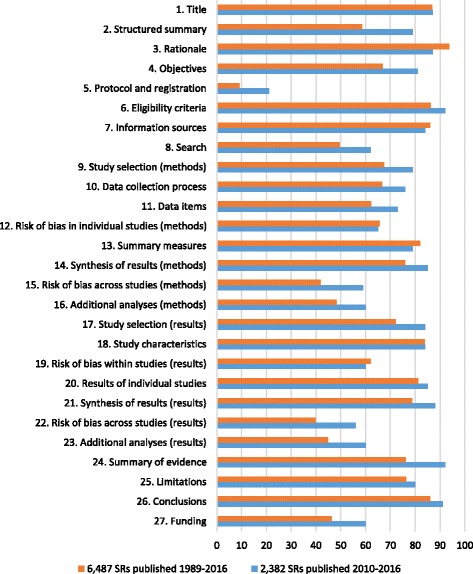
Summary percentage across reports of SRs adhering to the PRISMA Statement

All 57 studies assessed adherence to individual PRISMA items, with relevant data provided on request by authors of ten studies [39, 42, 43, 45, 66, 67, 69, 77, 79, 85]. By pooling the PRISMA adherence data across SRs in all 57 reports, we identified 11 items that fewer than 67% of SRs adhered to (Fig. 4; numerical data available in Additional file 3: Table S3). These include item 2 (structured summary), item 5 (methods: protocol and registration), item 8 (methods: search), item 11 (methods: data items), item 12 (methods: risk of bias in individual studies), item 15 (methods: risk of bias across studies), item 16 (methods: additional analyses), item 19 (results: risk of bias within studies), item 22 (results: risk of bias across studies), item 23 (results: additional analyses) and item 27 (funding). There were six items that fewer than 50% of SRs adhered to (items 5, 15, 16, 22, 23 and 27).

PRISMA adherence data for SRs published in 2010 or later (i.e. after the PRISMA Statement was published) were available in 27 studies [38, 39, 41, 42, 44, 46, 56, 60, 62, 68–79, 81–84, 92, 94], which evaluated 2382 SRs. The characteristics of these studies (i.e. focus, clinical area, language of SRs) were similar to those of the total set of studies. SR adherence to the PRISMA Statement was higher for nearly all items in this subset of recent SRs, compared with the adherence data across all SRs (Fig. 4; numerical data available in Additional file 3: Table S3). There were 12 items that more than 80% of SRs adhered to (items 1, 3, 4, 6, 7, 14, 17, 18, 20, 21, 24 and 26). However, lack of transparency remains an issue for many SRs. There were nine items that fewer than 67% of SRs adhered to (items 5, 8, 12, 15, 16, 19, 22, 23 and 27), and one item was adhered to by 21% of SRs only (item 5, on whether a SR protocol or registration number exists).

## Discussion

Our scoping review suggests that the PRISMA Statement and extensions have provided fertile ground for meta-research. Twenty different types of evidence were available across 100 meta-research studies. The most common type of evidence was data on SR adherence to the PRISMA Statement, which has been evaluated in 57 studies. The pooled results of these studies indicate that reporting of many items of the PRISMA Statement is suboptimal, even in those SRs published after its dissemination in 2009. Very few meta-research studies have evaluated SR adherence to the PRISMA extensions, but this is unsurprising given that most extensions were disseminated in 2015 or later. Few studies have tested strategies to increase adherence to the PRISMA Statement and extensions.

### Strengths and limitations

There are several strengths of our research. To our knowledge, this is the first attempt to systematically map research conducted on the PRISMA Statement and extensions. Most of the included studies assessing SR adherence to the PRISMA Statement focused on one clinical area, so by pooling data across these studies, our findings are more generalisable. Also, we managed to obtain unpublished data from ten studies that had not reported data on adherence to each individual PRISMA item [39, 42, 43, 45, 66, 67, 69, 77, 79, 85].

A few limitations must be acknowledged. We included only meta-research articles indexed in one bibliographic database (MEDLINE®) and written in English. However, we do not see any reason why our findings would differ had other databases and meta-research articles in languages other than English been consulted. Screening of records and collection of data from articles were performed by one author only. It is therefore possible that we may have missed some relevant meta-research studies or made errors when recording the frequency of SRs adhering to the PRISMA Statement. We have uploaded all data collected to the Open Science Framework (https://osf.io/7x2mp/) so that interested readers can verify our data and replicate our results. Most of the SRs evaluated in the 57 studies investigating SR adherence to the PRISMA Statement were written in English, and it is possible that non-English language SRs may be less likely to adhere to PRISMA, if their authors were not confident in English. Our classification of types of evidence available in meta-research studies reflects what was *reported*; we did not contact study authors to enquire whether they conducted other analyses yet chose not to report the findings. We did not record the references of SRs evaluated in each study investigating SR adherence to the PRISMA Statement and so are unaware if some SRs appeared in more than one of the included meta-research studies. However, based on the information regarding the types of SRs (e.g. Cochrane or non-Cochrane), years of publication of SRs and clinical focus of SRs, we judged the number of overlapping SRs to be low.

We were unable to compare the reporting of SRs published after PRISMA was disseminated in 2009 with that before 2009 because of how the included meta-research studies were designed and reported. Most studies (43 of 57) included some SRs published before 2009 and some published after 2009, but most studies did not report the number of SRs in each category. There were 14 studies that included only SRs published after 2009, 13 studies which provided subgroup data on SRs published after 2009 (but not all of these studies provided corresponding data for SRs published before 2009) and three studies included only SRs published before 2009. Given the data on PRISMA adherence in SRs published before 2009 was limited to a small subset of the included studies, we decided to restrict our analysis of PRISMA adherence to all SRs (regardless of year of publication) and SRs published after 2009. A formal before-after comparison was therefore not possible.

We focused on the PRISMA Statement and extensions, although we are aware of other reporting guidelines for SRs. These include the Methodological Expectations of Cochrane Intervention Reviews (MECIR) reporting standards [132, 133], the American Psychological Association Meta-Analysis Reporting Standards (MARS) [134], the ENTREQ Statement for syntheses of qualitative research [135], the RAMESES publication standards for realist syntheses [136] and meta-narrative reviews [137] and reporting guidance for describing interventions in SRs [138]. More research is needed to map the research conducted on these reporting guidelines.

### Comparison with other studies

We are aware of two other syntheses of meta-research studies that have investigated the adherence of SRs to the PRISMA Statement [35, 36]. Samaan et al. [36] included three studies, and Pussegoda et al. [35] included 13 studies, respectively. Both reached the same conclusion as us, that adherence to the PRISMA Statement is suboptimal; however, unlike our review, neither analysed reporting of SRs published after the PRISMA Statement was published. Another SR by Stevens et al. [37] synthesised the results of three studies exploring whether SR adherence to the PRISMA Statement is higher in journals which endorse the reporting guideline. We identified in our scoping review an additional five studies that could be added to an update of this review. To our knowledge, ours is the only review which has mapped research conducted on the PRISMA extensions.

### Implications of the findings

There are several reasons why adherence is better for some PRISMA items than others. It is possible that the less complex the item, the easier it is to report it. For example, most of the 12 PRISMA items that were adhered to by more than 80% of SRs published in 2010 or later are relatively straightforward to report. These items include identifying the report as a SR or meta-analysis in the title, providing a rationale and objectives, presenting study characteristics and reporting conclusions. Several items in the PRISMA Statement comprise multiple components, which some systematic reviewers may fail to fully address (e.g. item 12 asks authors to ‘describe methods used for assessing risk of bias of individual studies (including specification of whether this was done at the study or outcome level), and how this information is to be used in any data synthesis’). Also, reporting of some items may depend on whether the journal facilitates reporting of that item (e.g. authors may be unable to present a full electronic search strategy (item 8) in journals that do not allow supplementary files). In addition, some items with low adherence may not be considered sufficiently important to report by a majority of systematic reviewers and journal editors. It would be useful to conduct surveys and interviews with systematic reviewers to explore the contributions of these potential barriers and facilitators to complete SR reporting.

To our knowledge, there have been no prospectively designed, controlled studies evaluating whether the PRISMA Statement or extensions are having their intended effect. This is surprising, and a different threshold than that required to introduce a drug into the marketplace. Instead, only a few cross-sectional or uncontrolled before-after studies have evaluated the impact of journal endorsement of the PRISMA Statement on reporting of SRs. Of these eight studies [38, 39, 46, 71, 77, 81, 84, 94], six evaluated whether journals which ‘recommend’ or ‘encourage’ use of the PRISMA Statement in the journal instructions to authors publish SRs that are reported more completely. Two studies investigated whether reporting is clearer in journals that ask authors to submit a PRISMA checklist when submitting an SR. Both are rather low-intensity interventions that may not have the desired effect. For example, a recommendation in the instructions to authors can easily be missed by authors (some of whom will not even check the instructions), while a submitted PRISMA checklist may be ignored by peer reviewers and journal editors who face competing pressures on their time.

Researchers need to develop more efficient and intensive interventions to implement reporting guidelines such as the PRISMA Statement and extensions. We believe technology can play a valuable role in this regard. For example, StatReviewer software performs an automated review of the statistical and reporting integrity of scientific manuscripts (http://www.statreviewer.com/). Manuscripts can currently be checked against the following reporting guidelines: CONSORT 2010 [139], STROBE [140], STARD [141, 142], ARRIVE [143] and The Uniform Requirements for Medical Journals (http://www.icmje.org/recommendations/). StatReviewer is considering including PRISMA in their suite of reporting guidelines (D. Moher, personal communication). We also think rigorous evaluations, in the form of randomised trials, of StatReviewer are needed. Such evaluations could build upon the experiences of previous randomised trials evaluating web-based reporting guideline tools (e.g. WebCONSORT [144], COBWEB [145]).

It is 12 years since the PRISMA group last met, and the PRISMA Statement has not been updated since its publication 8 years ago. We believe that an update is necessary to address the poor adherence to the guideline. An updating process will provide the opportunity to discuss how to rearrange the layout and rephrase the checklist items to increase clarity. It will also allow for potential new items to be considered, based on recent methodological developments affecting SR conduct and reporting. These developments include novel guidance on how to:

- summarise findings when meta-analysis is not appropriate [146, 147];
- report and synthesise intervention characteristics of included studies [138, 148];
- use and interpret prediction intervals for random-effects meta-analyses [149, 150];
- enhance reproducibility of meta-analytic results and share data collected [151, 152] and
- report the methods and results of updated SRs [153] and living SRs [154].

In addition, developing a comprehensive research translation strategy to help journals endorse and implement the updated guideline may facilitate its use. Journal editors and researchers should work together to develop prospective (ideally randomised), controlled studies to provide robust evidence about the effect of the updated guideline on the transparency of SRs.

## Conclusions

Many studies have evaluated how well SRs adhere to the PRISMA Statement, and the pooled result of these suggests that reporting of many items is suboptimal. Little research has been done to design and test strategies to increase adherence to the PRISMA Statement or extensions. An update of the PRISMA Statement, followed by a toolkit of strategies to help journals endorse and implement the updated guideline, may improve the transparency of SRs.

## Additional files

Additional file 1: Tables of excluded papers. (DOCX 21 kb)

Additional file 2: List of included meta-research studies. (DOCX 25 kb)

Additional file 3: Summary across reports of SRs adhering to the PRISMA Statement. (DOCX 17 kb)

## Abbreviations

CI: Confidence interval
E&E: Explanation and elaboration
EQUATOR: Enhancing the QUAlity and Transparency Of health Research
IPD: Individual participant data
NMA: Network meta-analysis
PRISMA: Preferred Reporting Items for Systematic reviews and Meta-Analyses
QUOROM: QUality Of Reporting Of Meta-analyses
SR: Systematic review
