# Supplementary material for: Evaluations of the uptake and impact of the Preferred Reporting Items for Systematic reviews and Meta-Analyses (PRISMA) Statement and extensions: a scoping review
Source: Syst Rev. 2017 Dec 19;6:263. doi: 10.1186/s13643-017-0663-8 (PMC5738221; doi:10.1186/s13643-017-0663-8)
Supplement: Supplementary file 1 — Tables of excluded papers. (DOCX 21 kb) [file 13643_2017_663_MOESM1_ESM.docx]

**Additional file 1**

**Table S1. Table of excluded papers**

| **Citations of excluded papers** | **Reason for exclusion** |
| --- | --- |
| Antes G, von Elm E. [The PRISMA Statement - what should be reported about systematic reviews?]. Deutsche Medizinische Wochenschrift. 2009;134(33):1619. | Editorial/commentary |
| Barkun JS. Reply to letter: "meta-analyses, from GIGO to PRISMA". Annals of Surgery. 2015;261(5):e130-1. | Editorial/commentary |
| Beller EM, Glasziou PP, Altman DG, Hopewell S, Bastian H, Chalmers I, et al. PRISMA for Abstracts: reporting systematic reviews in journal and conference abstracts. PLoS Medicine / Public Library of Science. 2013;10(4):e1001419. | PRISMA Extension |
| Bernardo WM. PRISMA statement and PROSPERO. International Braz J Urol. 2017;43(3):383-4. | Editorial/commentary |
| Cornell JE. The PRISMA extension for network meta-analysis: bringing clarity and guidance to the reporting of systematic reviews incorporating network meta-analyses. Annals of Internal Medicine. 2015;162(11):797-8. | Editorial/commentary |
| Costa LO, Maher CG, Lopes AD, de Noronha MA, Costa LC. Transparent reporting of studies relevant to physical therapy practice. Revista Brasileira de Fisioterapia. 2011;15(4):267-71. | Editorial/commentary |
| Editors PM. Bringing clarity to the reporting of health equity. PLoS Medicine / Public Library of Science. 2012;9(10):e1001334. | Editorial/commentary |
| Erb HN. Changing expectations: Do journals drive methodological changes? Should they? Preventive Veterinary Medicine. 2010;97(3-4):165-74. | Editorial/commentary |
| Farid-Kapadia M, Askie L, Hartling L, Contopoulos-Ioannidis D, Bhutta ZA, Soll R, et al. Do systematic reviews on pediatric topics need special methodological considerations? BMC Pediatrics. 2017;17(1):57. | Editorial/commentary |
| Foster RL. Reporting guidelines: CONSORT, PRISMA, and SQUIRE. Journal for Specialists in Pediatric Nursing: JSPN. 2012;17(1):1-2. | Editorial/commentary |
| Gates NJ, March EG. A Neuropsychologist's Guide To Undertaking a Systematic Review for Publication: Making the most of PRISMA Guidelines. Neuropsychology Review. 2016;26(2):109-20. | Guidance/Tutorial |
| Glasziou P, Altman DG, Bossuyt P, Boutron I, Clarke M, Julious S, et al. Reducing waste from incomplete or unusable reports of biomedical research. Lancet. 2014;383(9913):267-76. | Editorial/commentary |
| Greenhalgh T, Wong G, Westhorp G, Pawson R. Protocol--realist and meta-narrative evidence synthesis: evolving standards (RAMESES). BMC Medical Research Methodology. 2011;11:115. | RAMESES protocol |
| Guise JM, Butler M, Chang C, Viswanathan M, Pigott T, Tugwell P, et al. AHRQ series on complex intervention systematic reviews-paper 7: PRISMA-CI elaboration and explanation. Journal of Clinical Epidemiology. 2017;15:15. | PRISMA Extension |
| Guise JM, Butler ME, Chang C, Viswanathan M, Pigott T, Tugwell P, et al. AHRQ Series on Complex Intervention Systematic Reviews - Paper 6: PRISMA-CI Extension Statement & Checklist. Journal of Clinical Epidemiology. 2017;28:28. | PRISMA Extension |
| Harms M. The EQUATOR Network and the PRISMA Statement for the reporting of systematic reviews and meta-analyses. Physiotherapy. 2009;95(4):237-40. | Editorial/commentary |
| Harris JD, Brand JC, Cote MP, Dhawan A. Research Pearls: The Significance of Statistics and Perils of Pooling. Part 3: Pearls and Pitfalls of Meta-analyses and Systematic Reviews. Arthroscopy. 2017;27:27. | Guidance/Tutorial |
| Harris JD, Quatman CE, Manring MM, Siston RA, Flanigan DC. How to write a systematic review. American Journal of Sports Medicine. 2014;42(11):2761-8. | Guidance/Tutorial |
| Hutton B, Catala-Lopez F, Moher D. [The PRISMA statement extension for systematic reviews incorporating network meta-analysis: PRISMA-NMA]. Medicina Clinica. 2016;147(6):262-6. | Editorial/commentary |
| Hutton B, Moher D, Cameron C. The PRISMA Extension Statement. Annals of Internal Medicine. 2015;163(7):566-7. | Editorial/commentary |
| Hutton B, Salanti G, Caldwell DM, Chaimani A, Schmid CH, Cameron C, et al. The PRISMA extension statement for reporting of systematic reviews incorporating network meta-analyses of health care interventions: checklist and explanations. Annals of Internal Medicine. 2015;162(11):777-84. | PRISMA Extension |
| Hutton B, Wolfe D, Moher D, Shamseer L. Reporting guidance considerations from a statistical perspective: overview of tools to enhance the rigour of reporting of randomised trials and systematic reviews. Evidence-Based Mental Health. 2017;20(2):46-52. | Editorial/commentary |
| Ishii LE. Optimizing Quality of Systematic Reviews in Plastic Surgery With the PRISMA Checklist. JAMA Facial Plastic Surgery. 2016;18(2):106-7. | Editorial/commentary |
| Kapadia MZ, Askie L, Hartling L, Contopoulos-Ioannidis D, Bhutta ZA, Soll R, et al. PRISMA-Children (C) and PRISMA-Protocol for Children (P-C) Extensions: a study protocol for the development of guidelines for the conduct and reporting of systematic reviews and meta-analyses of newborn and child health research. BMJ Open. 2016;6(4):e010270. | PRISMA Extension |
| Kearney MH. Hoping for a TREND toward PRISMA: the variety and value of research reporting guidelines. Research in Nursing & Health. 2014;37(2):85-7. | Editorial/commentary |
| Knobloch K, Yoon U, Vogt PM. Preferred reporting items for systematic reviews and meta-analyses (PRISMA) statement and publication bias. Journal of Cranio-Maxillo-Facial Surgery. 2011;39(2):91-2. | Editorial/commentary |
| Lachat C, Hodge A, Vandevijvere S, Villamor E, Tseng M. Introducing PRISMA as a requirement. Public Health Nutrition. 2015;18(14):2509-10. | Editorial/commentary |
| Liberati A, Altman DG, Tetzlaff J, Mulrow C, Gotzsche PC, Ioannidis JP, et al. The PRISMA statement for reporting systematic reviews and meta-analyses of studies that evaluate health care interventions: explanation and elaboration. Annals of Internal Medicine. 2009;151(4):W65-94. | PRISMA Statement |
| Liberati A, Altman DG, Tetzlaff J, Mulrow C, Gotzsche PC, Ioannidis JP, et al. The PRISMA statement for reporting systematic reviews and meta-analyses of studies that evaluate health care interventions: explanation and elaboration. Journal of Clinical Epidemiology. 2009;62(10):e1-34. | PRISMA Statement |
| Liberati A, Altman DG, Tetzlaff J, Mulrow C, Gotzsche PC, Ioannidis JP, et al. The PRISMA statement for reporting systematic reviews and meta-analyses of studies that evaluate health care interventions: explanation and elaboration. PLoS Medicine / Public Library of Science. 2009;6(7):e1000100. | PRISMA Statement |
| Liberati A, Altman DG, Tetzlaff J, Mulrow C, Gotzsche PC, Ioannidis JP, et al. The PRISMA statement for reporting systematic reviews and meta-analyses of studies that evaluate healthcare interventions: explanation and elaboration. BMJ. 2009;339:b2700. | PRISMA Statement |
| Maher C. PRISMA: helping to deliver information that physical therapists need. Physical Therapy. 2009;89(9):870-2. | Editorial/commentary |
| Mandrekar JN, Mandrekar SJ. Systematic reviews and meta-analysis of published studies: an overview and best practices. Journal of Thoracic Oncology: Official Publication of the International Association for the Study of Lung Cancer. 2011;6(8):1301-3. | Editorial/commentary |
| McLeroy KR, Northridge ME, Balcazar H, Greenberg MR, Landers SJ. Reporting guidelines and the American Journal of Public Health's adoption of Preferred Reporting Items for Systematic reviews and Meta-Analyses. American Journal of Public Health. 2012;102(5):780-4. | Editorial/commentary |
| Moher D, Altman DG, Liberati A, Tetzlaff J. PRISMA statement. Epidemiology. 2011;22(1):128; author reply | Editorial/commentary |
| Moher D, Liberati A, Tetzlaff J, Altman DG, Group P. Preferred reporting items for systematic reviews and meta-analyses: the PRISMA statement. Annals of Internal Medicine. 2009;151(4):264-9, W64. | PRISMA Statement |
| Moher D, Liberati A, Tetzlaff J, Altman DG, Group P. Preferred reporting items for systematic reviews and meta-analyses: the PRISMA statement. BMJ. 2009;339:b2535. | PRISMA Statement |
| Moher D, Liberati A, Tetzlaff J, Altman DG, Group P. Preferred reporting items for systematic reviews and meta-analyses: the PRISMA statement. Journal of Clinical Epidemiology. 2009;62(10):1006-12. | PRISMA Statement |
| Moher D, Liberati A, Tetzlaff J, Altman DG, Group P. Preferred reporting items for systematic reviews and meta-analyses: the PRISMA statement. PLoS Medicine / Public Library of Science. 2009;6(7):e1000097. | PRISMA Statement |
| Moher D, Liberati A, Tetzlaff J, Altman DG, Group P. Preferred reporting items for systematic reviews and meta-analyses: the PRISMA statement.[Erratum appears in Int J Surg. 2010;8(8):658]. International Journal Of Surgery. 2010;8(5):336-41. | PRISMA Statement |
| Moher D, Liberati A, Tetzlaff J, Altman DG, Group P. Reprint--preferred reporting items for systematic reviews and meta-analyses: the PRISMA statement. Physical Therapy. 2009;89(9):873-80. | PRISMA Statement |
| Moher D, Shamseer L, Clarke M, Ghersi D, Liberati A, Petticrew M, et al. Preferred reporting items for systematic review and meta-analysis protocols (PRISMA-P) 2015 statement. Systematic Reviews. 2015;4:1. | PRISMA Extension |
| Moher D, Stewart L, Shekelle P. Implementing PRISMA-P: recommendations for prospective authors. Systematic Reviews. 2016;5:15. | Editorial/commentary |
| Murad MH, Wang Z. Guidelines for reporting meta-epidemiological methodology research. Evidence Based Medicine. 2017;22(4):139-42. | Guidance/Tutorial |
| Page MJ, McKenzie JE, Kirkham J, Dwan K, Kramer S, Green S, et al. Bias due to selective inclusion and reporting of outcomes and analyses in systematic reviews of randomised trials of healthcare interventions. Cochrane Database of Systematic Reviews. 2014(10):MR000035. | Meta-research study without data relevant to PRISMA or its extensions |
| Peters MD. Managing and Coding References for Systematic Reviews and Scoping Reviews in EndNote. Medical Reference Services Quarterly. 2017;36(1):19-31. | Guidance/Tutorial |
| Sarkis-Onofre R, Cenci MS, Demarco FF, Lynch CD, Fleming PS, Pereira-Cenci T, et al. Use of guidelines to improve the quality and transparency of reporting oral health research. Journal of Dentistry. 2015;43(4):397-404. | Editorial/commentary |
| Schlattmann P, Schuetz GM, Dewey M. Wake up and smell the PRISMA, Cochrane, and QUADAS statements. Radiology. 2011;261(1):325-6; author reply 6-7. | Editorial/commentary |
| Shamseer L, Moher D, Clarke M, Ghersi D, Liberati A, Petticrew M, et al. Preferred reporting items for systematic review and meta-analysis protocols (PRISMA-P) 2015: elaboration and explanation. BMJ. 2015;349:g7647. | PRISMA Extension |
| Simera I. Get the content right: following reporting guidelines will make your research paper more complete, transparent and usable. JPMA - Journal of the Pakistan Medical Association. 2013;63(2):283-5. | Editorial/commentary |
| Sporbeck B, Jacobs A, Hartmann V, Nast A. Methodological standards in medical reporting. Journal der Deutschen Dermatologischen Gesellschaft. 2013;11(2):107-20. | Editorial/commentary |
| Stevanovic A, Coburn M, Rossaint R. [Minimum requirements for high quality reporting of medical research results : CONSORT, STROBE and PRISMA statements]. Anaesthesist. 2015;64(12):903-10. | Editorial/commentary |
| Stewart LA, Clarke M, Rovers M, Riley RD, Simmonds M, Stewart G, et al. Preferred Reporting Items for Systematic Review and Meta-Analyses of individual participant data: the PRISMA-IPD Statement. JAMA. 2015;313(16):1657-65. | PRISMA Extension |
| Stovold E, Beecher D, Foxlee R, Noel-Storr A. Study flow diagrams in Cochrane systematic review updates: an adapted PRISMA flow diagram. Systematic Reviews. 2014;3:54. | Editorial/commentary |
| Swartz MK. The PRISMA statement: a guideline for systematic reviews and meta-analyses. Journal of Pediatric Health Care. 2011;25(1):1-2. | Editorial/commentary |
| Thorlund K, Druyts E, Avina-Zubieta JA, Wu P, Mills EJ. Why the findings of published multiple treatment comparison meta-analyses of biologic treatments for rheumatoid arthritis are different: an overview of recurrent methodological shortcomings. Annals of the Rheumatic Diseases. 2013;72(9):1524-35. | Meta-research study without data relevant to PRISMA or its extensions |
| Tian JH, Ge L, Li L. The PRISMA Extension Statement. Annals of Internal Medicine. 2015;163(7):566. | Editorial/commentary |
| Tricco AC, Straus SE, Moher D. How can we improve the interpretation of systematic reviews? BMC Medicine. 2011;9:31. | Editorial/commentary |
| Urrutia G, Bonfill X. [PRISMA declaration: a proposal to improve the publication of systematic reviews and meta-analyses]. Medicina Clinica. 2010;135(11):507-11. | Editorial/commentary |
| Urrutia G, Bonfill X. [The PRISMA statement: a step in the improvement of the publications of the Revista Espanola de Salud Publica]. Revista Espanola de Salud Publica. 2013;87(2):99-102. | Editorial/commentary |
| Vandenbroucke JP. STREGA, STROBE, STARD, SQUIRE, MOOSE, PRISMA, GNOSIS, TREND, ORION, COREQ, QUOROM, REMARK... and CONSORT: for whom does the guideline toll? Journal of Clinical Epidemiology. 2009;62(6):594-6. | Editorial/commentary |
| Vrabel M. Preferred Reporting Items for Systematic Reviews and Meta-Analyses. Oncology Nursing Forum. 2015;42(5):552-4. | Editorial/commentary |
| Walther S, Schuetz GM, Hamm B, Dewey M. [Quality of reporting of systematic reviews and meta-analyses: PRISMA (Preferred Reporting Items for Systematic reviews and Meta-Analyses)]. Rofo: Fortschritte auf dem Gebiete der Rontgenstrahlen und der Nuklearmedizin. 2011;183(12):1106-10. | Editorial/commentary |
| Welch V, Petticrew M, Petkovic J, Moher D, Waters E, White H, et al. Extending the PRISMA statement to equity-focused systematic reviews (PRISMA-E 2012): explanation and elaboration. International Journal for Equity in Health. 2015;14:92. | PRISMA Extension |
| Welch V, Petticrew M, Petkovic J, Moher D, Waters E, White H, et al. Extending the PRISMA statement to equity-focused systematic reviews (PRISMA-E 2012): explanation and elaboration. Journal of Clinical Epidemiology. 2016;70:68-89. | PRISMA Extension |
| Welch V, Petticrew M, Tugwell P, Moher D, O'Neill J, Waters E, et al. PRISMA-Equity 2012 extension: reporting guidelines for systematic reviews with a focus on health equity. PLoS Medicine / Public Library of Science. 2012;9(10):e1001333. | PRISMA Extension |
| Welch VA, Petticrew M, O'Neill J, Waters E, Armstrong R, Bhutta ZA, et al. Health equity: evidence synthesis and knowledge translation methods. Systematic Reviews. 2013;2:43. | Guidance/Tutorial |
| Yavchitz A, Ravaud P, Hopewell S, Baron G, Boutron I. Impact of adding a limitations section to abstracts of systematic reviews on readers' interpretation: a randomized controlled trial. BMC Medical Research Methodology. 2014;14:123. | Meta-research study without data relevant to PRISMA or its extensions |
| Ziegler A, Konig IR. [Guidelines for research reports: German translation of CONSORT 2010, PRISMA and STARD]. Deutsche Medizinische Wochenschrift. 2011;136(8):357-8. | Editorial/commentary |
| Zorzela L, Loke YK, Ioannidis JP, Golder S, Santaguida P, Altman DG, et al. PRISMA harms checklist: improving harms reporting in systematic reviews.[Erratum appears in BMJ. 2016;353:i2229; PMID: 27094210]. BMJ. 2016;352:i157. | PRISMA Extension |
