# Supplementary material for: Evaluations of the uptake and impact of the Preferred Reporting Items for Systematic reviews and Meta-Analyses (PRISMA) Statement and extensions: a scoping review
Source: Syst Rev. 2017 Dec 19;6:263. doi: 10.1186/s13643-017-0663-8 (PMC5738221; doi:10.1186/s13643-017-0663-8)
Supplement: Supplementary file 2 — List of included meta-research studies. (DOCX 25 kb) [file 13643_2017_663_MOESM2_ESM.docx]

**Additional file 2**

**Table S2. List of included meta-research studies**

| **Citations of included studies** |
| --- |
| 1. Adie S, Ma D, Harris IA, Naylor JM, Craig JC. Quality of conduct and reporting of meta-analyses of surgical interventions. Annals of Surgery. 2015;261(4):685-94. |
| 1. Agha RA, Fowler AJ, Limb C, Whitehurst K, Coe R, Sagoo H, et al. Impact of the mandatory implementation of reporting guidelines on reporting quality in a surgical journal: A before and after study. International Journal Of Surgery. 2016;30:169-72. |
| 1. Aguiar PM, Brito GD, Correr CJ, Lyra Junior DP, Storpirtis S. Exploring the Quality of Systematic Reviews on Pharmacist Interventions in Patients With Diabetes: An Overview. The Annals of pharmacotherapy. 2014;48(7):887-96 |
| 1. Akhigbe T, Zolnourian A, Bulters D. Compliance of systematic reviews articles in brain arteriovenous malformation with PRISMA statement guidelines: Review of literature. Journal of Clinical Neuroscience. 2017;39:45-8. |
| 1. Atakpo P, Vassar M. Publication bias in dermatology systematic reviews and meta-analyses. Journal of Dermatological Science. 2016;82(2):69-74. |
| 1. Bigna JJ, Um LN, Nansseu JR. A comparison of quality of abstracts of systematic reviews including meta-analysis of randomized controlled trials in high-impact general medicine journals before and after the publication of PRISMA extension for abstracts: a systematic review and meta-analysis. Systematic Reviews. 2016;5(1):174. |
| 1. Bryce S, Sloan E, Lee S, Ponsford J, Rossell S. Cognitive remediation in schizophrenia: A methodological appraisal of systematic reviews and meta-analyses. Journal of Psychiatric Research. 2016;75:91-106. |
| 1. Burford BJ, Welch V, Waters E, Tugwell P, Moher D, O'Neill J, et al. Testing the PRISMA-Equity 2012 reporting guideline: the perspectives of systematic review authors. PLoS ONE . 2013;8(10):e75122. |
| 1. Campbell JM, Kavanagh S, Kurmis R, Munn Z. Systematic Reviews in Burns Care: Poor Quality and Getting Worse. Journal of Burn Care & Research. 2017;38(2):e552-e67. |
| 1. Chapman SJ, Drake TM, Bolton WS, Barnard J, Bhangu A. Longitudinal analysis of reporting and quality of systematic reviews in high-impact surgical journals. British Journal of Surgery. 2017;104(3):198-204. |
| 1. Chong AB, Taylor M, Schubert G, Vassar M. Interventional Radiology Clinical Practice Guideline Recommendations for Neurovascular Disorders Are Not Based on High-Quality Systematic Reviews. Ajnr: American Journal of Neuroradiology. 2017;38(4):759-65. |
| 1. Cullis PS, Gudlaugsdottir K, Andrews J. A systematic review of the quality of conduct and reporting of systematic reviews and meta-analyses in paediatric surgery. PLoS ONE . 2017;12(4):e0175213. |
| 1. DiSilvestro KJ, Tjoumakaris FP, Maltenfort MG, Spindler KP, Freedman KB. Systematic Reviews in Sports Medicine. American Journal of Sports Medicine. 2016;44(2):533-8. |
| 1. Evaniew N, van der Watt L, Bhandari M, Ghert M, Aleem I, Drew B, et al. Strategies to improve the credibility of meta-analyses in spine surgery: a systematic survey. Spine Journal: Official Journal of the North American Spine Society. 2015;15(9):2066-76. |
| 1. Farid-Kapadia M, Joachim KC, Balasingham C, Clyburne-Sherin A, Offringa M. Are child-centric aspects in newborn and child health systematic review and meta-analysis protocols and reports adequately reported?-two systematic reviews. Syst Rev. 2017;6(1):31. |
| 1. Fleming PS, Koletsi D, Pandis N. Blinded by PRISMA: are systematic reviewers focusing on PRISMA and ignoring other guidelines? PLoS ONE . 2014;9(5):e96407. |
| 1. Fleming PS, Koletsi D, Seehra J, Pandis N. Systematic reviews published in higher impact clinical journals were of higher quality. Journal of Clinical Epidemiology. 2014;67(7):754-9. |
| 1. Fleming PS, Seehra J, Polychronopoulou A, Fedorowicz Z, Pandis N. A PRISMA assessment of the reporting quality of systematic reviews in orthodontics. Angle Orthodontist. 2013;83(1):158-63. |
| 1. Gagnier JJ, Kellam PJ. Reporting and methodological quality of systematic reviews in the orthopaedic literature. Journal of Bone & Joint Surgery - American Volume. 2013;95(11):e771-7. |
| 1. Ge L, Tian JH, Li XX, Song F, Li L, Zhang J, et al. Epidemiology Characteristics, Methodological Assessment and Reporting of Statistical Analysis of Network Meta-Analyses in the Field of Cancer. Scientific Reports. 2016;6:37208. |
| 1. Ge L, Wang JC, Li JL, Liang L, An N, Shi XT, et al. The assessment of the quality of reporting of systematic reviews/meta-analyses in diagnostic tests published by authors in China. PLoS ONE . 2014;9(1):e85908. |
| 1. Gianola S, Gasparini M, Agostini M, Castellini G, Corbetta D, Gozzer P, et al. Survey of the reporting characteristics of systematic reviews in rehabilitation. Physical Therapy. 2013;93(11):1456-66. |
| 1. Glujovsky D, Boggino C, Riestra B, Coscia A, Sueldo CE, Ciapponi A. Quality of reporting in infertility journals. Fertility & Sterility. 2015;103(1):236-41. |
| 1. Glujovsky D, Villanueva E, Reveiz L, Murasaki R. [Adherence to research reporting guidelines in biomedical journals in Latin America and the Caribbean]. Pan American Journal of Public Health. 2014;36(4):232-7. |
| 1. Hammad TA, Neyarapally GA, Pinheiro SP, Iyasu S, Rochester G, Dal Pan G. Reporting of meta-analyses of randomized controlled trials with a focus on drug safety: an empirical assessment. Clinical Trials. 2013;10(3):389-97. |
| 1. Hedin RJ, Umberham BA, Detweiler BN, Kollmorgen L, Vassar M. Publication Bias and Nonreporting Found in Majority of Systematic Reviews and Meta-analyses in Anesthesiology Journals. Anesthesia & Analgesia. 2016;123(4):1018-25. |
| 1. Hirst A, Altman DG. Are peer reviewers encouraged to use reporting guidelines? A survey of 116 health research journals. PLoS ONE . 2012;7(4):e35621. |
| 1. Hua F, Walsh T, Glenny AM, Worthington H. Surveys on Reporting Guideline Usage in Dental Journals. Journal of Dental Research. 2016;95(11):1207-13. |
| 1. Hutton B, Salanti G, Chaimani A, Caldwell DM, Schmid C, Thorlund K, et al. The quality of reporting methods and results in network meta-analyses: an overview of reviews and suggestions for improvement. PLoS ONE . 2014;9(3):e92508. |
| 1. Jin YH, Ma ET, Gao WJ, Hua W, Dou HY. Reporting and methodological quality of systematic reviews or meta-analyses in nursing field in China. International Journal of Nursing Practice. 2014;20(1):70-8. |
| 1. Kelly SE, Moher D, Clifford TJ. Quality of conduct and reporting in rapid reviews: an exploration of compliance with PRISMA and AMSTAR guidelines. Systematic Reviews. 2016;5:79. |
| 1. Kiriakou J, Pandis N, Fleming PS, Madianos P, Polychronopoulou A. Reporting quality of systematic review abstracts in leading oral implantology journals. Journal of Dentistry. 2013;41(12):1181-7. |
| 1. Klimo P, Jr., Thompson CJ, Ragel BT, Boop FA. Methodology and reporting of meta-analyses in the neurosurgical literature. Journal of Neurosurgery. 2014;120(4):796-810. |
| 1. Knuppel H, Metz C, Meerpohl JJ, Strech D. How psychiatry journals support the unbiased translation of clinical research. A cross-sectional study of editorial policies. PLoS ONE . 2013;8(10):e75995. |
| 1. Koch M, Riss P, Umek W, Hanzal E. The explicit mentioning of reporting guidelines in urogynecology journals in 2013: A bibliometric study. Neurourology & Urodynamics. 2016;35(3):412-6. |
| 1. Kurz A, Evaniew N, Yeung M, Samuelsson K, Peterson D, Ayeni OR. Credibility and quality of meta-analyses addressing graft choice in anterior cruciate ligament reconstruction: a systematic review. Knee Surgery, Sports Traumatology, Arthroscopy. 2017;25(2):538-51. |
| 1. Lee AW. Use of network meta-analysis in systematic reviews: a survey of authors. Systematic Reviews. 2016;5:8. |
| 1. Lee SY, Sagoo H, Whitehurst K, Wellstead G, Fowler AJ, Agha RA, et al. Compliance of Systematic Reviews in Plastic Surgery With the PRISMA Statement. JAMA Facial Plastic Surgery. 2016;18(2):101-5. |
| 1. Li JL, Ge L, Ma JC, Zeng QL, Yao L, An N, et al. Quality of reporting of systematic reviews published in "evidence-based" Chinese journals. Systematic Reviews. 2014;3:58. |
| 1. Li X, Wang R, Shi X, Su J, Pan Y, Tian J, et al. Reporting Characteristics and Quality of Systematic Reviews of Acupuncture Analgesia. Pain Practice. 2017;13:13. |
| 1. Liu D, Jin J, Tian J, Yang K. Quality assessment and factor analysis of systematic reviews and meta-analyses of endoscopic ultrasound diagnosis. PLoS ONE . 2015;10(4):e0120911. |
| 1. Liu P, Qiu Y, Qian Y, Chen X, Wang Y, Cui J, et al. Quality of meta-analyses in major leading gastroenterology and hepatology journals: A systematic review. Journal of Gastroenterology & Hepatology. 2017;32(1):39-44. |
| 1. Liu X, Kinzler M, Yuan J, He G, Zhang L. Low Reporting Quality of the Meta-Analyses in Diagnostic Pathology. Archives of Pathology & Laboratory Medicine. 2017;141(3):423-30. |
| 1. Liu Y, Zhang R, Huang J, Zhao X, Liu D, Sun W, et al. Reporting quality of systematic reviews/meta-analyses of acupuncture. PLoS ONE . 2014;9(11):e113172. |
| 1. Luo J, Xu H, Yang G, Qiu Y, Liu J, Chen K. Oral Chinese proprietary medicine for angina pectoris: an overview of systematic reviews/meta-analyses. Complementary Therapies in Medicine. 2014;22(4):787-800. |
| 1. Ma B, Guo J, Qi G, Li H, Peng J, Zhang Y, et al. Epidemiology, quality and reporting characteristics of systematic reviews of traditional Chinese medicine interventions published in Chinese journals. PLoS ONE . 2011;6(5):e20185. |
| 1. Ma B, Qi GQ, Lin XT, Wang T, Chen ZM, Yang KH. Epidemiology, quality, and reporting characteristics of systematic reviews of acupuncture interventions published in Chinese journals. Journal of Alternative & Complementary Medicine. 2012;18(9):813-7. |
| 1. Mannocci A, Saulle R, Colamesta V, D'Aguanno S, Giraldi G, Maffongelli E, et al. What is the impact of reporting guidelines on Public Health journals in Europe? The case of STROBE, CONSORT and PRISMA. Journal of Public Health. 2015;37(4):737-40. |
| 1. Martins DE, Astur N, Kanas M, Ferretti M, Lenza M, Wajchenberg M. Quality assessment of systematic reviews for surgical treatment of low back pain: an overview. Spine Journal: Official Journal of the North American Spine Society. 2016;16(5):667-75. |
| 1. McGee RG, Craig JC, Rogerson TE, Webster AC. Systematic reviews of surgical procedures in children: quantity, coverage and quality. Journal of paediatrics and child health. 2013;49(4):319-24. |
| 1. Meerpohl JJ, Wolff RF, Antes G, von Elm E. Are pediatric Open Access journals promoting good publication practice? An analysis of author instructions. BMC Pediatrics. 2011;11:27. |
| 1. Nicolau I, Ling D, Tian L, Lienhardt C, Pai M. Methodological and reporting quality of systematic reviews on tuberculosis. International Journal of Tuberculosis & Lung Disease. 2013;17(9):1160-9. |
| 1. Nissen T, Wayant C, Wahlstrom A, Sinnett P, Fugate C, Herrington J, et al. Methodological quality, completeness of reporting and use of systematic reviews as evidence in clinical practice guidelines for paediatric overweight and obesity. Clinical Obesity. 2017;7(1):34-45. |
| 1. Padula RS, Pires RS, Alouche SR, Chiavegato LD, Lopes AD, Costa LO. Analysis of reporting of systematic reviews in physical therapy published in Portuguese. Revista Brasileira de Fisioterapia. 2012;16(4):381-8. |
| 1. Page MJ, Shamseer L, Altman DG, Tetzlaff J, Sampson M, Tricco AC, et al. Epidemiology and Reporting Characteristics of Systematic Reviews of Biomedical Research: A Cross-Sectional Study. PLoS Med. 2016;13(5):e1002028. |
| 1. Panic N, Leoncini E, de Belvis G, Ricciardi W, Boccia S. Evaluation of the endorsement of the preferred reporting items for systematic reviews and meta-analysis (PRISMA) statement on the quality of published systematic review and meta-analyses. PLoS ONE . 2013;8(12):e83138. |
| 1. Passon AM, Drabik A, Sawicki PT. Quality scores do not predict discrepant statistical significances among meta-analyses on different targets of glycemic control in type 2 diabetes. Journal of Clinical Epidemiology. 2013;66(12):1356-66. |
| 1. Pastorino R, Milovanovic S, Stojanovic J, Efremov L, Amore R, Boccia S. Quality Assessment of Studies Published in Open Access and Subscription Journals: Results of a Systematic Evaluation. PLoS ONE . 2016;11(5):e0154217. |
| 1. Peters JP, Hooft L, Grolman W, Stegeman I. Reporting Quality of Systematic Reviews and Meta-Analyses of Otorhinolaryngologic Articles Based on the PRISMA Statement. PLoS ONE . 2015;10(8):e0136540. |
| 1. Pidgeon TE, Wellstead G, Sagoo H, Jafree DJ, Fowler AJ, Agha RA. An assessment of the compliance of systematic review articles published in craniofacial surgery with the PRISMA statement guidelines: A systematic review. Journal of Cranio-Maxillo-Facial Surgery. 2016;44(10):1522-30. |
| 1. Pinzon MC, Hayden DM, Ariel D, Bartosiak KA, Chiodo MV, Kosmidis K, et al. Are our publications failing the inspection?: a review of the publications in rectal cancer surgery between 2002 and 2012. Diseases of the Colon & Rectum. 2014;57(8):983-92. |
| 1. Pussegoda K, Turner L, Garritty C, Mayhew A, Skidmore B, Stevens A, et al. Identifying approaches for assessing methodological and reporting quality of systematic reviews: a descriptive study. Systematic Reviews. 2017;6(1):117. |
| 1. Pussegoda K, Turner L, Garritty C, Mayhew A, Skidmore B, Stevens A, et al. Systematic review adherence to methodological or reporting quality. Systematic Reviews. 2017;6(1):131. |
| 1. Rader T, Mann M, Stansfield C, Cooper C, Sampson M. Methods for documenting systematic review searches: a discussion of common issues. Research Synthesis Methods. 2014;5(2):98-115. |
| 1. Reveiz L, Villanueva E, Iko C, Simera I. Compliance with clinical trial registration and reporting guidelines by Latin American and Caribbean journals. Cadernos de Saude Publica. 2013;29(6):1095-100. |
| 1. Riado Minguez D, Kowalski M, Vallve Odena M, Longin Pontzen D, Jelicic Kadic A, Jeric M, et al. Methodological and Reporting Quality of Systematic Reviews Published in the Highest Ranking Journals in the Field of Pain. Anesthesia and analgesia. 2017. |
| 1. Riaz IB, Khan MS, Riaz H, Goldberg RJ. Disorganized Systematic Reviews and Meta-analyses: Time to Systematize the Conduct and Publication of These Study Overviews? American Journal of Medicine. 2016;129(3):339.e11-8. |
| 1. Rice DB, Kloda LA, Shrier I, Thombs BD. Reporting completeness and transparency of meta-analyses of depression screening tool accuracy: A comparison of meta-analyses published before and after the PRISMA statement. Journal of psychosomatic research. 2016;87:57-69. |
| 1. Saltaji H, Ospina MB, Armijo-Olivo S, Agarwal S, Cummings GG, Amin M, et al. Evaluation of risk of bias assessment of trials in systematic reviews of oral health interventions, 1991-2014: A methodology study. Journal of the American Dental Association. 2016;147(9):720-8.e1. |
| 1. Samaan Z, Mbuagbaw L, Kosa D, Borg Debono V, Dillenburg R, Zhang S, et al. A systematic scoping review of adherence to reporting guidelines in health care literature. Journal of multidisciplinary healthcare. 2013;6:169-88. |
| 1. Scott J, Howard B, Sinnett P, Schiesel M, Baker J, Henderson P, et al. Variable methodological quality and use found in systematic reviews referenced in STEMI clinical practice guidelines. American Journal of Emergency Medicine. 2017;14:14. |
| 1. Seehra J, Fleming PS, Polychronopoulou A, Pandis N. Reporting completeness of abstracts of systematic reviews published in leading dental specialty journals. European Journal of Oral Sciences. 2013;121(2):57-62. |
| 1. Shi C, Zhu L, Wang X, Qin C, Xu Q, Tian J. Epidemiology, methodological and reporting characteristics of systematic reviews of nursing interventions published in China. International Journal of Nursing Practice. 2014;20(6):689-700. |
| 1. Shi X, Wang X, Liu Y, Li X, Wei D, Zhao X, et al. A survey of evidence users about the information need of acupuncture clinical evidence. BMC Complementary & Alternative Medicine. 2016;16(1):455. |
| 1. Simmonds M, Stewart G, Stewart L. A decade of individual participant data meta-analyses: A review of current practice. Contemporary Clinical Trials. 2015;45(Pt A):76-83. |
| 1. Sims MT, Henning NM, Wayant CC, Vassar M. Do emergency medicine journals promote trial registration and adherence to reporting guidelines? A survey of "Instructions for Authors". Scandinavian Journal of Trauma, Resuscitation & Emergency Medicine. 2016;24(1):137. |
| 1. Smith TA, Kulatilake P, Brown LJ, Wigley J, Hameed W, Shantikumar S. Do surgery journals insist on reporting by CONSORT and PRISMA? A follow-up survey of 'instructions to authors'. Annals of Medicine & Surgery. 2015;4(1):17-21. |
| 1. Stevens A, Shamseer L, Weinstein E, Yazdi F, Turner L, Thielman J, et al. Relation of completeness of reporting of health research to journals' endorsement of reporting guidelines: systematic review. BMJ. 2014;348:g3804. |
| 1. Tam WW, Lo KK, Khalechelvam P. Endorsement of PRISMA statement and quality of systematic reviews and meta-analyses published in nursing journals: a cross-sectional study. BMJ Open. 2017;7(2):e013905. |
| 1. Tan WK, Wigley J, Shantikumar S. The reporting quality of systematic reviews and meta-analyses in vascular surgery needs improvement: a systematic review. International Journal Of Surgery. 2014;12(12):1262-5. |
| 1. Tao KM, Li XQ, Zhou QH, Moher D, Ling CQ, Yu WF. From QUOROM to PRISMA: a survey of high-impact medical journals' instructions to authors and a review of systematic reviews in anesthesia literature. PLoS ONE . 2011;6(11):e27611. |
| 1. Tian J, Zhang J, Ge L, Yang K, Song F. The methodological and reporting quality of systematic reviews from China and the USA are similar. J Clin Epidemiol. 2017;85:50-8. |
| 1. Toews I, Binder N, Wolff RF, Toprak G, von Elm E, Meerpohl JJ. Guidance in author instructions of hematology and oncology journals: A cross sectional and longitudinal study. PloS one. 2017;12(4):e0176489. |
| 1. Toews LC. Compliance of systematic reviews in veterinary journals with Preferred Reporting Items for Systematic Reviews and Meta-Analysis (PRISMA) literature search reporting guidelines. Journal of the Medical Library Association. 2017;105(3):233-9. |
| 1. Tsou AY, Treadwell JR. Quality and clarity in systematic review abstracts: an empirical study. Research Synthesis Methods. 2016;7(4):447-58. |
| 1. Tsujimoto Y, Tsujimoto H, Kataoka Y, Kimachi M, Shimizu S, Ikenoue T, et al. Majority of systematic reviews published in high-impact journals neglected to register the protocols: a meta-epidemiological study. Journal of Clinical Epidemiology. 2017;84:54-60. |
| 1. Tunis AS, McInnes MD, Hanna R, Esmail K. Association of study quality with completeness of reporting: have completeness of reporting and quality of systematic reviews and meta-analyses in major radiology journals changed since publication of the PRISMA statement?.[Erratum appears in Radiology. 2014 Jul;272(1):304]. Radiology. 2013;269(2):413-26. |
| 1. Turner L, Galipeau J, Garritty C, Manheimer E, Wieland LS, Yazdi F, et al. An evaluation of epidemiological and reporting characteristics of complementary and alternative medicine (CAM) systematic reviews (SRs). PLoS ONE . 2013;8(1):e53536. |
| 1. van der Pol CB, McInnes MD, Petrcich W, Tunis AS, Hanna R. Is quality and completeness of reporting of systematic reviews and meta-analyses published in high impact radiology journals associated with citation rates? PLoS ONE . 2015;10(3):e0119892. |
| 1. Wasiak J, Shen AY, Ware R, O'Donohoe TJ, Faggion CM, Jr. Methodological quality and reporting of systematic reviews in hand and wrist pathology. Journal of Hand Surgery: European Volume. 2017:1753193417712660. |
| 1. Wasiak J, Tyack Z, Ware R, Goodwin N, Faggion CM, Jr. Poor methodological quality and reporting standards of systematic reviews in burn care management. International Wound Journal. 2016;18:18. |
| 1. Wayant C, Smith C, Sims M, Vassar M. Hematology journals do not sufficiently adhere to reporting guidelines: a systematic review. Journal of Thrombosis & Haemostasis. 2017;15(4):608-17. |
| 1. Weir CR, Staggers N, Laukert T. Reviewing the impact of computerized provider order entry on clinical outcomes: The quality of systematic reviews. International Journal of Medical Informatics. 2012;81(4):219-31. |
| 1. Willis BH, Quigley M. The assessment of the quality of reporting of meta-analyses in diagnostic research: a systematic review. BMC Medical Research Methodology. 2011;11:163. |
| 1. Xiao Z, Zhang Y, Wang Y, Xu F. Quality assessment for systematic review/meta-analysis on antidepressant therapy published in Chinese journals. International Journal of Pharmacology. 2012;8(7):614-20. |
| 1. Yang M, Jiang L, Wang A, Xu G. Epidemiology characteristics, reporting characteristics, and methodological quality of systematic reviews and meta-analyses on traditional Chinese medicine nursing interventions published in Chinese journals. International Journal of Nursing Practice. 2017;23(1). |
| 1. Yang SL, Ying K, Wang F, Wang L, Ren XY, Yang QF. [Methodological and reporting quality assessment for Chinese systematic reviews and meta analysis in oral medicine]. Shanghai Kou Qiang Yi Xue/Shanghai Journal of Stomatology. 2015;24(4):505-10. |
| 1. Zhang H, Han J, Zhu YB, Lau WY, Schwartz ME, Xie GQ, et al. Reporting and methodological qualities of published surgical meta-analyses. Journal of Clinical Epidemiology. 2016;70:4-16. |
| 1. Zhang J, Wang J, Han L, Zhang F, Cao J, Ma Y. Epidemiology, quality, and reporting characteristics of systematic reviews and meta-analyses of nursing interventions published in Chinese journals. Nursing Outlook. 2015;63(4):446-55.e4. |
| 1. Zhu Y, Fan L, Zhang H, Wang M, Mei X, Hou J, et al. Is the Best Evidence Good Enough: Quality Assessment and Factor Analysis of Meta-Analyses on Depression. PLoS ONE . 2016;11(6):e0157808. |
